# Hepatocellular carcinoma and the penetrance of *HFE *C282Y mutations: a cross sectional study

**DOI:** 10.1186/1471-230X-5-17

**Published:** 2005-06-01

**Authors:** Gavin Willis, Vicky Bardsley, Ian W Fellows, Ray Lonsdale, Jennie Z Wimperis, Barbara A Jennings

**Affiliations:** 1 Department of Molecular Genetics, Norfolk and Norwich University Hospital, Norwich, NR47UY, UK; 2 Department of Histopathology, Norfolk and Norwich University Hospital, Norwich, NR47UY, UK; 3 Department of Gastroenterology, Norfolk and Norwich University Hospital, Norwich, NR47UY, UK; 4 Department of Haematology, Norfolk and Norwich University Hospital, Norwich, NR47UY, UK; 5 School of Medicine, Health Policy and Practice, University of East Anglia, Norwich, NR47PT, UK

## Abstract

**Background:**

Although most patients with hereditary haemochromatosis have *HFE *C282Y mutations, the lifetime risk to *HFE *C282Y homozygotes of developing fatal diseases such as hepatocellular carcinoma is uncertain. We have carried out a cross-sectional study to determine the proportion of diagnosed hepatocellular carcinoma patients who are homozygous for the *HFE *C282Y mutation; and to estimate the penetrance of this genotype with respect to hepatocellular carcinoma in East Anglia.

**Methods:**

Tissue biopsies were analysed from 144 cases of hepatocellular carcinoma for *HFE *C282Y mutations; the data produced were compared with the frequency of *HFE *mutations in a large sample of the local population. Data were also retrieved from the East Anglian Cancer Intelligence Unit to determine the annual incidence of hepatocellular carcinoma; and from appropriate life tables.

**Results:**

Eight out of 144 of the cases were homozygous for the *HFE *C282Y mutation, all 8 cases were male. 6 of these 8 cases had a previous diagnosis of hereditary haemochromatosis. Male *HFE *C282Y homozygotes were more likely to be diagnosed with hepatocellular carcinoma (odds ratio [OR] = 14, 95% confidence interval [CI] = 5–37). For this population, we estimate that the penetrance of the *HFE *C282Y homozygous genotype, with respect to hepatocellular carcinoma, was between 1.31 % and 2.1% for males and was zero for females.

**Conclusion:**

In this population, we found that only a very small proportion of homozygotes for the *HFE *C282Y mutation developed hepatocellular carcinoma. However, individuals with this genotype have a significantly increased risk of this rare disease relative to those who do not carry the mutations.

## Background

Hereditary haemochromatosis is an autosomal recessive genetic condition in which excess iron is absorbed by the intestine. Individuals with the clinical manifestations of the disease (which include liver cirrhosis, hepatocellular carcinoma, diabetes mellitus, cardiomyopathy and arthropathy) will have accumulated iron over many years of adult life resulting in progressive tissue damage. Liver disease is the commonest cause of death of patients with hereditary haemochromatosis [1,2]. A recent cohort study of patients diagnosed with haemochromatosis in Sweden found that at ten years follow-up, the absolute risk of liver cancer was 6% among men and 1.5% among women [3]. This patient cohort is likely to be at higher risk of liver cancer than those *HFE *C282Y homozygotes who do not display the signs and symptoms of haemochromatosis [3].

Haemochromatosis is unusual among genetic diseases because it can easily be treated. Individuals diagnosed and treated by regular venesection before symptoms of cirrhosis occur have a normal life expectancy [4]. The discovery of mutations in the *HFE *gene that are present in most haemochromatosis patients has provided a useful test in families affected by the disease [5]. Two *HFE *genotypes are commonly associated with haemochromatosis: homozygosity for the C282Y (845A) mutation and compound heterozygosity with the C282Y and H63D (187G) mutation [5-7].

The high frequency of *HFE *mutations in the normal population and the availability of a treatment for haemochromatosis led to suggestions that population screening for these mutations would be justified on the basis of both health and cost [8,9]. The value of screening depends on the penetrance of the *HFE *genotypes. Penetrance in this case can be defined as the frequency with which individuals of particular genotypes develop either iron overload or clinical manifestations.

We have previously studied the penetrance of the *HFE *mutations with respect to haemochromatosis disease manifestations by comparing the predicted birth rate of *HFE *C282Y homozygotes in our study population of 500,000 with the incidence of *HFE *C282Y-homozygous patients diagnosed with haemochromatosis, cirrhosis [10], liver cancer [11], arthritis [12] or diabetes [13]. We showed that, in this large population, few *HFE *C282Y homozygotes (1.4%) were diagnosed with haemochromatosis and of the remainder few were diagnosed with liver disease (2.7% – 8%) or diabetes (0 – 1.3%).

Our findings suggested an unexpectedly low figure for penetrance which made our estimates for the potential benefit of genetic screening marginal.

Because of the need for more data to inform decisions about the value of population screening we have now collected a much larger cohort of hepatocellular carcinoma (HCC) cases (n = 144) for *HFE *genotyping. The samples were drawn from histology archives, collected over a thirty year period, from the two largest hospitals in East Anglia. We have used genotyping data from this cross-section of 144 cases and HCC incidence data from the cancer registry to deduce the incidence of *HFE *C282Y homozygotes diagnosed with HCC annually. We have compared this with the proportion of the general population who are *HFE *C282Y homozygotes and who reach the at-risk age for HCC annually; this corresponds to the penetrance of *HFE *C282Y homozygosity with respect to developing HCC.

## Methods

### Patient samples; hepatocellular carcinoma cases

The Norfolk and Norwich University Hospital has a catchment area of 500,000 people. 41 suitable HCC cases were identified from histopathology records from 1974 to 2001 regardless of any previous diagnosis of haemochromatosis. 29 cases were from men and 12 were from women. 28 of these 41 cases from Norwich were included in our previous studies [10,11].

Addenbrooke's Hospital, Cambridge, is a tertiary referral centre for hepatic transplantation and referral. Therefore, cases of liver cancer derive from the hospital's catchment area of approximately 350,000 individuals and from other hospitals in East Anglia, and less commonly from the rest of the U.K. and Italy. For this study, cases were rejected if the patient's name was Italian to exclude tertiary referral cases of southern European origin (because of differences in the prevalence of *HFE *mutations). 103 HCC cases from Addenbrooke's Hospital were identified by searching files from 1969 to 2000 for cases of HCC regardless of any previous diagnosis of haemochromatosis and included both biopsy cases and explanted livers. 73 cases were from men and 30 from women.

None of the 103 cases from Addenbrooke's Hospital had been analysed in the cohort of cases from the Norfolk and Norwich University Hospital.

Slides from all 144 cases from Norfolk and Cambridge were reviewed and the presence of HCC confirmed.

### Analysis of *HFE *genotype

Formalin-fixed, paraffin-wax embedded specimens were retrieved from the histology archives. DNA was extracted from 10 mm^2 ^of 10 μM tissue sections by sequential octane and acetone extractions; followed by digestion overnight at 55°C in 50 μl of 500 μg/ml proteinase K, 10 mM Tris, 1 mM EDTA and 0.5% Tween 20 and subsequent incubation for 10 minutes at 96°C.

Analysis of the *HFE *codon 282 genotypes was carried out on the PE Biosystems 7700 (Applied Biosystems), using standard allelic discrimination assay software and Taqman Universal PCR Mastermix (Applied Biosystems). In most cases, 1 μl of template DNA (approximately 50 ng) was used per assay, the primers and probes are described in table 1. The annealing temperature in a standard PE Biosystems 7700 protocol for the assay was 65°C. Four each of CY heterozygote and CC and YY homozygote and no DNA controls were included on each 96 well plate. The primers used amplify a 106 base pair fragment of the *HFE *gene and therefore the assay is suitable even for relatively degraded DNA.

### Population data

The control *HFE *genotype data (see table 2) discussed in this paper are pooled from two recent publications looking at *HFE *mutations in the Norfolk and Cambridgeshire populations [12,14].

The number of C282Y homozygotes reaching the at-risk age for HCC annually was calculated using data in the 1985–1988 life tables [15].

The annual incidence of HCC in East Anglia between 1970 and 2001 was obtained from the East Anglian Intelligence Unit (cancer registry) [16].

### Ethical approval

The analysis of the previously archived tumour and DNA samples was carried out with local research ethics committee (LREC) approval from Norwich (NDEC97/090) and Cambridge (LREC 00/153). All genetic analysis was carried out on anonymous samples.

### Statistical analysis

95% confidence intervals (CI) are exact binomial or, for ratios, the normal approximation; p values are by Fisher's exact test.

## Results

### Analysis of the *HFE *genotypes

8/144 (5.6 %, 95% CI = 2.4–10.7%) of the cases of HCC were homozygous for the *HFE *C282Y mutation. 102 of the 144 cases were males and all *HFE *C282Y homozygous cases were male. Thus 8/102 male HCC cases were *HFE *C282Y homozygotes while 9/1508 of the control population had this genotype (see table 2). Male *HFE *C282Y homozygotes were therefore more likely to be diagnosed with HCC (OR = 14, 95% CI = 5–37).

The 8 C282Y homozygous cases are described in table 3. Five of the *HFE *C282Y homozygous cases were from Cambridge; all these had been diagnosed previously with haemochromatosis. The three other *HFE *C282Y homozygous cases were from Norwich. One of these cases had been diagnosed with haemochromatosis. The remaining two cases had not been previously diagnosed with haemochromatosis. Because Addenbrooke's Hospital is a tertiary referral centre the details of the Norwich *HFE *C282Y homozygotes were checked against the Cambridge samples to ensure that there was no duplication.

17/144 (11.8%) cases were heterozygous for the *HFE *C282Y mutation. This frequency is essentially the same as that in the normal population (see table 2).

### Population data

#### C282Y homozygotes reaching the at-risk age for HCC annually

54 was chosen as the at-risk age for HCC because it is the mean age for presentation with HCC in our cohort. The proportion of the male population reaching the age of 54 annually (1260 per 100,000) was calculated from the birth rate (1390 per 100,000) and the proportion surviving to the age of 54 (lx = 0.912) in the 1985–1988 life tables [15]. The proportion of the population who are C282Y homozygotes reaching the age of 54 annually was calculated from the above figure multiplied by the observed proportion of C282Y homozygotes in the normal population: 7.5 per 100,000 [12,14].

#### The penetrance of the C282Y homozygous genotype with respect to HCC

The mean annual incidence of HCC in East Anglia between 1971 and 2001 was 1.26 per 100,000 males [16]. Our sample allows us to estimate that 7.8% (8 /102 males in the study) of these were *HFE *C282Y homozygotes (0.099 per 100,000). This figure contrasts with the proportion of the population who are C282Y homozygotes reaching the at-risk age for HCC annually: 7.5 per 100,000 (see above).

We thus estimate that only 1.31% (95% CI = 0.52–3.32%) of males homozygous for the *HFE *C282Y genotype are diagnosed and recorded with HCC in this population. This corresponds to an estimate of penetrance if most HCC cases are recorded by the cancer registry.

An alternative method to estimate the normal population frequency of C282Y homozygosity is to calculate the square of the C282Y allele frequency (0.062^2 ^= 0.0038). This figure has a lower standard error, being derived from the larger number of heterozygotes, but does not take account of population effects such as non-random mating or mixing. Repeating the above calculation using this approach gives an estimate for penetrance of 2.1% (95% CI = 0.89–4.05%).

In this study we have failed to see any penetrance for the C282Y homozygous genotype with respect to HCC for females.

### Histology

Slides for cases that were *HFE *C282Y homozygotes were reanalysed to examine the background liver abnormalities in more detail (see table 3). Two of the five C282Y homozygous samples from Addenbrooke's Hospital were biopsy specimens, and the remaining three were explanted livers. Two cases showed significant fibrosis with prominent linking of many portal tracts by fibrous bands, but did not show established cirrhosis. The remaining 3 cases all showed micronodular cirrhosis. These cases were staged for fibrosis using the method described by Ishak *et.al. *[17].

The needle biopsies of 3 C282Y homozygotes from Norwich were analysed, one biopsy consisted of tumour only, a second showed a minute area of cirrhotic liver and a third showed minimal fibrosis and was not cirrhotic; however, there was very little background liver tissue in this specimen.

All *HFE *C282Y homozygous cases with an adequate amount of background liver showed siderosis of grades 1 to 4, with or without a history of venesection treatment (see table 3).

## Discussion

The discovery of the *HFE *gene in 1996, the high prevalence of C282Y mutations, and the morbidity and mortality associated with untreated hereditary haemochromatosis have presented molecular diagnostics with a potentially attractive test for population screening.

Our results show that male *HFE *C282Y homozygotes were more likely to be diagnosed with HCC (OR = 14, 95% CI = 5–37); the *HFE *C282Y homozygous genotype could therefore be a significant cause of liver cancer. This is consistent with the results of longitudinal studies of haemochromatosis patient cohorts showing that primary liver cancer is a common cause of death [1-3]. We found 8 *HFE *C282Y homozygotes in a cohort of 144 HCC cases; of these 6 had been previously diagnosed with hereditary haemochromatosis. The genetic data from the 2 other cases could be interpreted as evidence of undiagnosed hereditary haemochromatosis leading to HCC. However, the clinical implication of this finding is uncertain because these cases were diagnosed with HCC in 1985 and 1990; before which there was less awareness of, and surveillance for, hereditary haemochromatosis.

Our previous studies have shown that most people with *HFE *mutations can survive to old age and do not suffer from signs of iron overload and haemochromatosis [18,19]. Large population screens also suggest that only a minority of *HFE *C282Y homozygotes develop clinical signs and symptoms of iron overload [20-22].

We have now studied a large cohort of HCC patients collected over three decades in a well-defined population. We estimate that between 1.31% (95% CI = 0.52–3.32%) and 2.1% (95% CI = 0.89–4.05%) of males homozygous for the C282Y genotype have diagnosed and recorded HCC. We found zero penetrance for the *HFE *C282Y homozygous genotype with respect to HCC in women. We have previously shown that, in Norfolk, only a small proportion of *HFE *C282Y homozygotes have been diagnosed with and treated with venesection for haemochromatosis [23]; therefore pre-cirrhotic management of haemochromatosis does not explain the low penetrance described.

These estimates of penetrance in men and women are higher and lower respectively than our previous combined estimate for men and women of 0.4% (95% CI = 0–1%) [11]. A combined figure for men and women based on the data presented here would be slightly higher than our previous estimate. This difference results mainly from a lower normal population frequency for the *HFE *C282Y allele in the much larger and better age-matched normal control population presented here.

To estimate the penetrance of the *HFE *mutations we have carried out a cross-sectional study of histologically confirmed cases of HCC and used cancer registry data that is reliant on an accurate clinical diagnosis. The main source of systematic error in estimating penetrance in this study is likely to be unrecorded or mis-classified HCC. If HCC cases have not been reported to the cancer registry or were not accurately classified (e.g. recorded as liver cancer not otherwise specified) then our calculation of the penetrance of these *HFE *mutations would be an under-estimate.

Any error is likely to be small for two reasons. First, cancer is a notifiable disease and, 18 months after diagnosis in East Anglia, ascertainment for all tumours is nearly 100% complete (Sara Godward, East Anglian Cancer Intelligence Unit; personal communication). Secondly, HCC usually develops as a long-term complication of cirrhosis which will often have been detectable several years beforehand.

The frequency of *HFE *C282Y homozygosity in HCC patients in this study (5.6%) is similar to those seen in other studies of northern European populations [24,25]. Blanc *et al. *found that 5.7% of individuals, in a selected group of French HCC cases developing without cirrhosis, were *HFE *C282Y homozygotes [24]. Cauza *et al. *found that 3.1% of a cross-section of 162 Austrian HCC cases were *HFE *C282Y homozygotes [25]. For males, Austria is an area with high rates of HCC (10.5/100,000) [26], low *HFE *C282Y allele frequencies (5%) [27,28] and similar life expectancy relative to Britain. Using this limited data we calculate that this corresponds to a higher penetrance, at about 10%, of *HFE *C282Y homozygosity with respect to HCC in Austria.

Studies from Italy [29,30] and Spain [31] have reported no *HFE *C282Y homozygotes among cohorts of HCC patients. However, these were small studies in populations with low *HFE *C282Y prevalence. One large study also found no *HFE *C282Y homozygotes among a cohort of French HCC patients [32]. However, the case ascertainment was different to our own; having excluded any patients suspected to have genetic haemochromatosis thus precluding comparison with our data.

The expression of the life-threatening clinical manifestations of haemochromatosis has been shown to be affected by environmental modifying factors that may also be population specific. Italian haemochromatosis patients who were older than 55 years, had cirrhosis, a history of high alcohol consumption and were positive for antibodies for hepatitis B at diagnosis, had a 150 times higher relative risk of HCC [33]. Excessive alcohol consumption was also shown to accentuate the expression of haemochromatosis in French *HFE *C282Y homozygotes [34]. One limitation of this study is that we did not collect data on any environmental risk factors that our patients with HCC were exposed to; such as high alcohol use and chronic viral hepatitis. The low mean annual incidence of HCC (1.26 per 100,000 males) in East Anglia over the last three decades could reflect low exposure to environmental risk factors.

The value of population screening for *HFE *C282Y mutations partly depends on the penetrance with respect to the life-threatening manifestations of haemochromatosis. The zero penetrance described in this study for female *HFE *C282Y homozygotes developing HCC is at odds with proposals for whole population screening. However, male *HFE *C282Y homozygotes have a high relative risk of developing HCC. A targeted screening strategy that considers synergistic factors could prove effective for the prevention of this fatal disease, on the grounds of both cost and health.

## Conclusion

• In this U.K. population we have shown that male *HFE *C282Y homozygotes are over-represented in a cross-section of confirmed HCC cases collected over three decades. Most of the *HFE *C282Y homozygotes had been previously diagnosed with hereditary haemochromatosis; we therefore found little evidence of undiagnosed haemochromatosis-related HCC over a thirty year period.

• We have used our genotyping data to estimate that between 1.31% and 2.1% of males homozygous for the C282Y genotype but no females have diagnosed and recorded HCC which corresponds to a low estimate of penetrance.

• The annual incidence of hepatocellular carcinoma in East Anglia is low relative to other populations which may reflect low exposure to environmental risk factors for HCC. In other populations these environmental risk factors have been shown to synergise with *HFE *mutations.

## Abbreviations

Hepatocellular carcinoma (HCC), odds ratio (OR) confidence interval (CI).

## Competing interests

The author(s) declare that they have no competing interests.

## Authors' contributions

GW and BAJ conceived of the study, participated in the design of the study*, carried out the genetic analysis and drafted the manuscript. GW also carried out the statistical analysis.

JZW and IWF participated in the design of the study* and helped to draft the document.

VB and RL participated in the design of the study*, sample collection, helped to draft the document and carried out histological analysis.

*The study design included LREC and research governance applications.

All authors read and approved the final manuscript.

## Pre-publication history

The pre-publication history for this paper can be accessed here:



## Figures and Tables

**Table 1:** HFE C282Y allelic discrimination assay using taqman probes. The primers and probes used to amplify and detect the portion of the *HFE *gene around codon 282.

	**Primers and Probes**	**Concentration**
**forward primer**	ggctggataaccttggctgtac	300 nM
**reverse primer**	gtcacataccccagatcacaatga	900 nM
**mutant probe**	fam tgctccacctggtacgtatatctctgct	100 nM
**wild type probe**	vic ctccacctggcacgtatatctctgct	100 nM

**Table 2:** *HFE *genotypes for codon 282 of HCC cases and control cohorts. Ages in years and numbers (percentages) of cases with each genotype.

	**HCC Patients**	Controls *	
	
**Mean Age**	54 ^a^	57	
**CC (normal)**	119 (82.6%)	1331 (88.2%)	
**CY (heterozygotes)**	17 (11.8%)	168 (11.1%)	p = 0.34
**YY (homozygotes)**	8 (5.6%)	9 (0.6%)	p = 0.00003
**Total Number**	144	1508	

p values are single tailed probabilities of over-representation of mutant genotypes in HCC patients by Fisher's exact test.

* Norfolk data presented in Willis *et al. *2002 [12], Cambridge data presented in Halsall *et.al. *2003 [14].

^a ^age data available for only 138/144 cases.

**Table 3:** Features of *HFE *C282Y homozygotes. Cases 1–3 were from Norwich, cases 1 and 2 were included in our previous study [11].

	**Histology (and histological staging of fibrosis)**	**Grade of Siderosis (liver iron)**	**Venesection^a^**	**Age#**	**Sex**
**1**	HCC (inadequate for staging)	NA*	No	66	M
**2**	HCC, cirrhosis (inadequate for staging)	Grade 1	No	70	M
**3**	HCC (inadequate for staging)	Grade 3/4	Yes	64	M
**4**	HCC, fibrosis (stage 4)	Grade 2/3	Yes	64	M
**5**	HCC, cirrhosis (stage 6)	Grade 4	No	73	M
**6**	HCC, fibrosis (stage 4)	Grade 1	Yes	64	M
**7**	HCC, cirrhosis (stage 6)	Grade 4	Not known	63	M
**8**	HCC, cirrhosis (stage 6)	Grade 2/3	Yes	62	M

^a ^Information about previous venesection treatment obtained from histology request forms and reports.

# Age at biopsy.

* Not applicable; only tumour was present in the needle biopsy sample.

HCC, hepatocellular carcinoma.

## References

[B1] Niederau C, Fischer R, Purschel A, Stremmel W, Haussinger D, Strohmeyer G (1996). Long term survival in patients with hereditary haemochromatosis. Gastroenterology.

[B2] Fracanzani AL, Conte D, Fraquelli M, Taioli E, Mattioli M, Losco A, Fargion S (2001). Increased cancer risk in a cohort of 230 patients with hereditary hemochromatosis in comparison to matched control patients with non-iron-related chronic liver disease. Hepatology.

[B3] Elmberg M, Hultcrantz R, Ekbom A, Brandt L, Olsson S, Olsson R, Lindgren S, Loof L, Stal P, Wallerstedt S, Almer S, Sandberg-Gertzen H, Askling J (2003). Cancer risk in patients with hereditary hemochromatosis and in their first-degree relatives. Gastroenterolology.

[B4] Niederau C, Fischer R, Sonnenberg A, Stremmel W, Trampisch HJ, Strohmeyer G (1985). Survival and causes of death in cirrhotic and in non cirrhotic patients with primary haemochromatosis. N Engl J Med.

[B5] Feder JN, Gnirke A, Thomas W, Tsuchihashi Z, Ruddy DA, Basava A, Dormishian F, Domingo R, Ellis MC, Fullan A, Hinton LM, Jones NL, Kimmel BE, Kronmal GS, Lauer P, Lee VK, Loeb DB, Mapa FA, McClelland E, Meyer NC, Mintier GA, Moeller N, Moore T, Morikang E, Prass CE, Quintana SM, Schatzman RC, Brunke KJ, Drayna DT, Risch NJ, Bacon BR, Wolff RK (1996). A novel MHC class I-like gene is mutated in patients with hereditary hemochromatosis. Nature Genet.

[B6] Beutler E, Gelbart T, West C, Lee P, Adams M, Blackstone R, Pockros P, Kosty M, Venditti CP, Phatak PD, Seese NK, Chorney KA, Ten Elshof AE, Gerhard GS, Chorney M (1996). Mutation analysis in hereditary hemochromatosis. Blood Cells Mol Dis.

[B7] The UK Haemochromatosis Consortium (1997). A simple genetic test identifies 90% of UK patients with haemochromatosis. Gut.

[B8] Bassett ML, Leggett BA, Halliday JW, Webb S, Powell LW (1997). Analysis of the cost of population screening for haemochromatosis using biochemical and genetic markers. Hepatology.

[B9] Burt MJ, George PM, Upton JD, Collett JA, Frampton CM, Chapman TM, Walmsley TA, Chapman BA (1998). The significance of haemochromatosis gene mutations in the general population: implications for screening. Gut.

[B10] Willis G, Wimperis JZ, Lonsdale R, Fellows IW, Watson MA, Skipper LM, Jennings BA (2000). Incidence of liver disease in people with HFE mutations. Gut.

[B11] Willis G, Wimperis JZ, Lonsdale R, Jennings BA (1997). Haemochromatosis gene mutation in hepatocellular cancer. Lancet.

[B12] Willis G, Scott DGI, Jennings BA, Smith K, Bukhari M, Wimperis JZ (2002). *HFE *mutations in an inflammatory arthritis population. Rheumatology.

[B13] Sampson MJ, Williams T, Heyburn PJ, Greenwood RH, Temple RC, Wimperis JZ, Jennings BA, Willis GA (2000). HFE Prevalence of HFE Haemochromatosis gene mutations in unselected male patients with Type 2 diabetes. J Lab Clin Med.

[B14] Halsall DJ, McFarlane I, Luan J, Cox TM, Wareham NJ (2003). Typical type 2 diabetes mellitus and HFE gene mutations: a population-based case-control study. Hum Mol Genet.

[B15] Government Actuary's Department Life tables. http://www.gad.gov.uk/Life_Tables/Interim_life_tables.htm.

[B16] East Anglian Cancer Intelligence Unit. http://www.phpc.cam.ac.uk/ciu/.

[B17] Ishak K, Baptista A, Bianchi L, Callea F, De Groote J, Gudat F, Denk H, Desmet V, Korb G, MacSween RNM, Phillips MJ, Portmann BG, Poulsen H, Scheuer PJ, Schmid M, Thaler H (1995). Histological grading and staging of chronic hepatitis. J Hepatol.

[B18] Willis G, Wimperis JZ, Smith KC, Fellows IW, Jennings BA (1999). HFE (haemochromatosis gene) C282Y homozygotes in an elderly male population. Lancet.

[B19] Willis G, Wimperis JZ, Smith K, Fellows IW, Jennings BA (2003). HFE mutations in the elderly. Blood Cells Mol Dis.

[B20] Jackson HA, Carter K, Darke C, Guttridge MG, Ravine D, Hutton RD, Napier JA, Worwood M (2001). HFE mutations, iron deficiency and overload in 10500 blood donors. Br J Haematol.

[B21] Beutler E, Felitti VJ, Koziol JA, Ho NJ, Gelbart T (2002). Penetrance of 845G→A (C282Y) HFE hereditary haemochromatosis mutation in the USA. Lancet.

[B22] Andersen RV, Tybjaerg-Hansen A, Appleyard M, Birgens H, Nordestgaard BG (2004). Hemochromatosis mutations in the general population: iron overload progression rate. Blood.

[B23] Willis G, Jennings BA, Goodman E, Fellows IW, Wimperis JZ A high prevalence of HLA-H 845A mutations in hemochromatosis patients and the normal population in eastern England. Blood Cells Mol Dis.

[B24] Blanc JF, De Ledinghen V, Bernard PH, de Verneuil H, Winnock M, Le Bail B, Carles J, Saric J, Balabaud C, Bioulac-Sage P (2000). Increased incidence of HFE C282Y mutations in patients with iron overload and hepatocellular carcinoma developed in non-cirrhotic liver. J Hepatol.

[B25] Cauza E, Peck-Radosavljevic M, Ulrich-Pur H, Datz C, Gschwantler M, Schoniger-Hekele M, Schoniger-Hekele M, Hackl F, Polli C, Rasoul-Rockenschaub S, Muller C, Wrba F, Gangl A, Ferenci P (2003). Mutations of the HFE gene in patients with hepatocellular carcinoma. Am J Gastroenterol.

[B26] Bray F, Sankila R, Ferlay J, Parkin DM (2002). Estimates of cancer incidence and mortality in Europe in 1995. Eur J Cancer.

[B27] Kazemi-Shirazi L, Datz C, Maier-Dobersberger T, Kaserer K, Hackl F, Polli CT, Steindl PE, Penner E, Ferenci P (1999). The relation of iron status and hemochromatosis gene mutations in patients with chronic hepatitis C. Gastroenterology.

[B28] Datz C, Haas T, Rinner H, Sandhofer F, Patsch W, Paulweber B (1998). Heterozygosity for the C282Y mutation in the hemochromatosis gene is associated with increased serum iron, transferring saturation, and haemoglobin in young women: a protective role against iron deficiency?. Clin Chem.

[B29] Racchi O, Mangerini R, Rapezzi D, Gaetani GF, Nobile MT, Picciotto A, Ferraris AM (1999). Mutations of the HFE gene and the risk of hepatocellular carcinoma. Blood Cells Mol Dis.

[B30] Pirisi M, Toniutto P, Uzzau A, Fabris C, Avellini C, Scott C, Apollonio L, Beltrami CA, Bresadola F (2000). Carriage of HFE mutations and outcome of surgical resection for hepatocellular carcinoma in cirrhotic patients. Cancer.

[B31] Lauret E, Rodriguez M, Gonzalez S, Linares A, Lopez-Vazquez A, Martinez-Borra J, Rodrigo L, Lopez-Larrea C (2002). HFE gene mutations in alcoholic and virus-related cirrhotic patients with hepatocellular carcinoma. Am J Gastroenterol.

[B32] Boige L, Castera L, de Roux N, Ganne-Carrie N, Ducot B, Pelletier G, Beaugrand M, Buffet C (2003). Lack of association between HFE gene mutations and hepatocellular carcinoma in patients with cirrhosis. Gut.

[B33] Fargion S, Fracanzani AL, Piperno A, Braga M, D'Alba R, Ronchi G, Fiorelli G (1994). Prognostic factors for hepatocellular carcinoma in genetic hemochromatosis. Hepatology.

[B34] Scotet V, Merour MC, Mercier AY, Chanu B, Le Faou T, Raguenes O, Le Gac G, Mura C, Nousbaum JB, Ferec C (2003). Hereditary hemochromatosis: effect of excessive alcohol consumption on disease expression in patients homozygous for the C282Y mutation. Am J Epidemiol.

